# Patient Satisfaction With the Head and Neck Cancer Telephone Triage Service During the COVID-19 Pandemic

**DOI:** 10.7759/cureus.18375

**Published:** 2021-09-29

**Authors:** Yinan Zhu, Zehong Chen, Anni Ding, Hannah Walter, Rachel Easto, Adam Wilde

**Affiliations:** 1 Otolaryngology - Head and Neck Surgery, University College Hospital, London, GBR; 2 Trauma and Orthopaedics, Sandwell and West Birmingham NHS Trust, Birmingham, GBR; 3 Otolaryngology, Derriford Hospital, Plymouth, GBR; 4 Otolaryngology, University of Plymouth, Plymouth, GBR; 5 Otolaryngology, Torbay Hospital, Torquay, GBR; 6 Otolaryngology - Head and Neck Surgery, Torbay Hospital, Torquay, GBR

**Keywords:** two week wait, patients satisfaction, covid-19 telemedicine, telephone consultation, head and neck neoplasms

## Abstract

Background

A telephone triage consultation, as part of the two-week wait head and neck cancer referral pathway, was implemented nationally in March 2020. This was in response to the COVID-19 pandemic to stream cancer referrals to minimize unnecessary interactions and appointments with health services. The aim of this study is to assess patient satisfaction with this novel telephone triage system in the setting of a district general hospital.

Methods

A custom designed patient satisfaction questionnaire covering different facets of the patient experience was used. These questions were adapted from several internally validated questionnaires. A retrospective telephone survey was conducted by interviewers for all continuous new head and neck cancer referrals over two 4-week periods in 2020. Questionnaire responses to the initial modality of consult (either telephone triage or face to face) were collected, and data were analysed both qualitatively and quantitatively.

Results

Seventy-five responses were received, with 51 patients providing feedback on an initial telephone triage consultation. Patients rated the telephone triage consultation to be between satisfied and very satisfied across most domains, with an overall score of 4.29 out of 5. Accessibility and efficiency of the telephone triage were the domains with the least satisfaction. Fifty-five percent of patients would be happy to receive a similar telephone triage consultation beyond the pandemic. Qualitative analysis showed praise for the safety and convenience of the telephone triage consultation during the pandemic but highlighted a general preference for a face-to-face consultation and dissatisfaction regarding a lack of physical examination.

Conclusions

Overall, patients are satisfied with the telephone triage consultation employed in the pandemic, with high satisfaction rates for multiple aspects of care. However, there were concerns regarding the accessibility and inefficiency associated with a lack of/delayed physical examination and inability to adequately address the fear and anxiety associated with the referral. A mixed response is obtained on whether the telephone triage system should stay for the long run.

## Introduction

The COVID-19 virus outbreak was declared a pandemic on 11th March 2020 by the World Health Organisation (WHO) [1]. This is justified by its alarming levels of spread and severity in a global context. Implementation of targeted, noncoercive community mitigation plans is critical to delay the exponential spread of the virus [2]. Social distancing is one such important measure to decrease the frequency and duration of social contact to reduce person-to-person transmission of the virus [2], particularly in socially dense indoor environments including hospitals. This is hugely relevant for the workup of head and neck cancers, as the COVID-19 virus predominantly resides in the airway, with high viral titres in the nasal and oral cavity [3]. It is transmitted between people through respiratory droplets and contact routes, particularly with aerosol generating procedures (AGP) [4]. Many outpatient head and neck examinations and investigations fall under the AGP umbrella. Consequently, since the pandemic began, ENT departments have had to consider who and how they perform these investigations to manage risk. The National Health Service (NHS) strategies for service provision in the diagnosis and treatment of cancer recommend an initial telephone appointment to stream cancer referrals [5].

As such, a head and neck cancer telephone triage pathway was developed by ENT UK and implemented in March 2020 using the evidence-based, structured head and neck cancer risk calculator (HaNC-RC)-V.2 [6]. It would replace an outpatient face-to-face clinic appointment as the first patient consultation upon referral. This involves asking 16 questions which include the patient’s age, gender, alcohol and smoking status, as well as the presence or absence of 12 significant symptoms via a telephone consultation [7]. At the end of this, the clinician would recommend an outcome based on the results of the risk assessment. This could mean booking the patient in for an urgent face-to-face appointment, scheduling a nonurgent appointment or discharging the patient directly. A structured suggested script is also published by ENT UK to aid the delivery of the telephone triage [8]. Despite the telephone triage being a relatively novel intervention for suspected head and neck cancer patients, an evaluation of this service at eight weeks has shown initial success, with 1,568 calls documented across the United Kingdom and 54.7% of these avoiding an urgent hospital visit during the peak of the pandemic [9].

Patient satisfaction stems from patient experience and, as best explained by the NHS published "The Patient Experience Book," is what the process of receiving care feels like for the patient, their family and carers [10]. This is the way in which care is delivered to achieve patient needs and "results in an environment where individual patients feel cared for and supported" [10]. This is a key element contributing to the quality provision of healthcare, alongside providing clinical excellence and safer care [10]. Satisfaction in a telephone consultation is a multidimensional concept [11], including both relational aspects such as being valued as an individual and functional aspects such as efficient clinical processes. The most pertinent themes elaborated in "What matters to patients?," an NHS quality improvement project developing an evidence base for the improvement of patient experience [12], will be used to guide our study.

The aim of this study is to assess multiple dimensions of patient satisfaction with the telephone triage consultation via a custom patient satisfaction questionnaire and appreciate underlying themes for satisfaction or dissatisfaction. Current planned studies explore the technical aspects of the telephone triage system including the efficacy of the existing risk calculators [9]. Our study will complement this with regards to the nontechnical facets of this novel system.

## Materials and methods

Data collection

All continuous new two-week wait (2WW) head and neck cancer referrals to Torbay Hospital since the inception of the telephone triage system from March 2020 to April 2020, as well as the month of August 2020, were included in the study. Two study periods are necessary to allow comparison between patients who had the telephone triage consultation at the peak of the pandemic versus those who had the traditional face-to-face consultations which resumed towards the end of the first wave. Following study approval, the patient list was obtained from the local hospital information team. Three authors (YZ, HW, AD) phoned patients with a suggested script to introduce the study before asking the 13 questions from the questionnaire. These questions were directed at the initial modality of consult, either via telephone triage (TT group) or a face-to-face consultation (F2F group). Participation was entirely voluntary. Responses were documented in a shared secure, confidential electronic database.

The protocol of this study and all questions used in the questionnaire have been approved by the Clinical Psychology team, as well as the Clinical Audit and Effectiveness Group of Torbay Hospital. Ethics approval was not required for this service evaluation project.

Analysis

Quantitative data were analysed using Prism (GraphPad, 9.2.0, San Diego, USA). All responses on the Likert scale were converted to a numerical scale from 1 (strongly disagree) to 5 (strongly agree). Patient satisfaction was also quantitatively inferred from this scale (1 to 5 representing very dissatisfied, dissatisfied, neutral, satisfied, very satisfied in this order). Descriptive statistics were computed (mean, standard deviation, ranges) for patient demographics (age, gender) as well as questionnaire answers. Unpaired T-test analysis was used to compare the mean values of each question answered by the TT and F2F groups. A two-tailed p-value of <0.05 was considered to be significant with 95% confidence intervals.

Qualitatively, open-ended questionnaire responses are inductively and thematically analysed. Key phrases are coded and then grouped into overarching themes [13], which are used to explain support or concerns with regards to the telephone triage system. 

Selection and rationale of questions used

The overall questionnaire was formulated from five different internally validated instruments including the Telemedicine Satisfaction Questionnaire (TSQ) [14], Visit-Specific Satisfaction Questionnaire (VSQ) [15], ﻿15-item Picker Patient Experience Questionnaire (PPE) [16], Generic Short Patient Experiences Questionnaire (GS-PEQ) [17] and the short form of the Quality from the Patient’s Perspective (QPP) questionnaire [18]. Questions (Q) 1-11 uses the five-point Likert scale. Q1 assesses the technical similarity of a telephone consultation to that of its face-to-face counterpart, Q2 gauges the patient’s perception of the interaction and Q3 measures accessibility of this healthcare service. Q4-6 assessed the relational aspects of care and personal mannerisms of the specialist in receiving and communicating information. Q7 assessed time spent during the consultation, Q8 information received, Q9 respect for patient preferences and Q10 the patient perceived effectiveness of the modality of consult. Q11 seeks an overall opinion. Q12-13 do not use the Likert scale, with Q12 being a polar interrogative and Q13 open ended. 

The authors felt that it was important to include an amalgamation of questions from the various established questionnaires and modify these to fit our unique context. This is so that multiple domains of patient satisfaction can be targeted while questions remain relevant. In fact, it is encouraged for such surveys to be specific and applicable in the evaluation of a service in question, using other questions/instruments if necessary [16-18]. In addition, the collection of qualitative data helps to triangulate findings as per data analysis recommendations [10]. 

The list of questions asked is presented in Table 1, along with the linked patient satisfaction themes identified from the NHS.

**Table 1:** Custom patient satisfaction questionnaire used with their original sources credited TSQ: Telemedicine Satisfaction Questionnaire, VSQ: Visit-Specific Satisfaction Questionnaire, QPP: Quality from the Patient’s Perspective Questionnaire, PPE: 15-item Picker Patient Experience Questionnaire, GS-PEQ: Generic Short Patient Experiences Questionnaire. Patient satisfaction themes from Q3 to Q9 derived from "What matters to patients?" [12].

No.	Question	Aspect of patient satisfaction	Source
1	My specialist is able to understand my healthcare concerns	Technical aspects: similarity to a face-to-face consultation	TSQ
2	I felt comfortable communicating with my specialist	Patient perception of a telephone consultation	TSQ
3	I obtain better access to healthcare services	Accessibility to healthcare services	TSQ
4	The specialist was friendly, courteous and respectful towards me	Good relationships and positive attitudes among staff	VSQ, QPP, PPE
5	The specialist showed concerns for my questions or worries	Being treated as a person, not a number	QPP
6	The specialist talked with me in a way that was easy to understand	Using language that is easy to understand	PPE, GS-PEQ
7	The specialist spent an adequate amount of time with me	Staff who listen and spend time with patient	VSQ
8	Information: The specialist gave explanations about my problem or condition	Feeling informed, receiving information and being given options	VSQ, GS-PEQ
9	The specialist included me in decisions about my treatment	Patient involvement in care and being able to ask questions	QPP, PPE, GS-PEQ
10	I find this consultation to be an acceptable way to address my health concerns	Effectiveness of consult	TSQ
11	Overall, I am satisfied with the quality of service being provided	Overall satisfaction	TSQ, VSQ
12	I would be happy to receive a phone consultation for the same health issues beyond the COVID-19 pandemic	-	Original
13	(Open ended) Any other positive or negative feedback regarding the phone consultation	-	Original

## Results

Seventy-five responses were collated out of 131 patients contacted (57.3% response rate). In total, there were a total of 27 males and 48 females, with an age range from 21 to 91 (mean: 58.7). Of which, 51 patients were in the TT group, while 24 were in the F2F group. Demographics for each group are presented in Table 2.

**Table 2:** Demographics of the telephone triage and face-to-face consultation groups

Demographics	Initial telephone triage group (n=51)	Initial face-to-face group (n=24)
Mean age (years)	58.8	58.4
Gender (n (%))		
Male	15 (29.4%)	12 (50%)
Female	36 (70.6%)	12 (50%)

Quantitative assessment

For Q1-11, patient satisfaction was between satisfied and very satisfied (4-5) for all domains for both the TT and F2F groups, except for Q3 (accessibility to healthcare services) and Q10 (effectiveness of consult for the TT group), in which patients were between neutral and satisfied (3-4) about. Comparatively, there is a significant difference in the above questions between the two groups (p<0.05), favouring the F2F group. These results are presented in Table 3.

**Table 3:** Comparison of the quantitative questionnaire results of the telephone triage and face-to-face consultation groups with unpaired T-tests

No.	Question theme	Initial telephone triage group (n=51), mean (SD)	Initial face to face group (n=24), mean (SD)	T-test p-value
1	Technical aspects: similarity to a face-to-face consultation	4.41 (0.73)	4.42 (0.58)	0.98
2	Patient perception of a telephone consultation	4.45 (0.61)	4.58 (0.58)	0.38
3	Accessibility to healthcare services	3.67 (1.07)	4.29 (0.69)	0.01
4	Good relationships and positive attitudes among staff	4.71 (0.46)	4.79 (0.41)	0.44
5	Being treated as a person, not a number	4.57 (0.50)	4.63 (0.58)	0.67
6	Using language that is easy to understand	4.53 (0.54)	4.75 (0.44)	0.09
7	Staff who listen and spend time with patient	4.45 (0.58)	4.58 (0.65)	0.38
8	Feeling informed, receiving information and being given options	4.33 (0.65)	4.58 (0.65)	0.13
9	Patient involvement in care and being able to ask questions	4.18 (0.78)	4.50 (0.78)	0.11
10	Effectiveness of consult	3.63 (1.11)	4.75 (0.44)	<0.01
11	Overall satisfaction	4.29 (0.88)	4.54 (0.78)	0.24
12	Satisfaction to receive a phone consultation beyond the pandemic	Yes: 28 (55%) No: 23 (45%)	Yes: 10 (42%) No: 14 (58%)	-

Qualitative assessment

Thematic analysis produced four main themes. These are presented in Table 4.

**Table 4:** Themes identified from the thematic analysis of the open-ended question for all questionnaire participants

No.	Theme	Elaboration
1	Telephone triage lacks immediate physical examination: Delays diagnosis and hinders communication	35 responses mentioned the importance of physical examination as a key component lacking in the initial telephone triage
		12 responses: Delays diagnosis: “No examination, no amount of talking was able to assess, still needed to wait for face to face anyway”
		5 responses: Hinders communication with clinician: “was much harder to describe over the phone than to show the doctor where the problem was”
2	Doctor-patient relationship: Better with face-to-face consultation	24 responses mentioned the relational aspects of the consultation, including confidence, communication, empathy, etc. While the vast majority (20) favoured the face-to-face consult, a few (4) were satisfied with the telephone triage
		Preference for face to face: “Like to have a face and speak to them, don’t want to be talking over the phone,” “Over the phone you lose the personable bit,” “physical contact, facial expressions important, empathy better face to face”
		Happy with telephone triage: “Found the telephone consultation I had was very much a human touch and informative”
3	Better access to care during pandemic: Telephone consultation safe, easier accessibility and cost savings	19 responses praised the telephone triage service for its safety during the pandemic, ease of access and cost savings
		Patient appreciation of pandemic factors: “Understand pandemic and burden on healthcare needs,” “frees up the consultants”
		Ease and cost savings: “Saves travel time, car parking charges,” “Child care much easier with telephone consultation,” “Patient housebound so telephone consultation was a very accessible way to receive healthcare”
		6 neutral/negative responses: “have a car not a problem," “still have to wait for examination,” “wait for clinic appointment anyway, waste of time”
4	Emotional aspect: Telephone triage may induce more fear and anxiety	9 responses spoke about the emotional aspects of the consultation. While some (2) were reassured by the telephone triage, more (5) felt increased fear/anxiety post call.
		Telephone triage increased fear/anxiety: “I paid to go privately so that I could see a doctor face-to-face for an examination,” “Can be a pain when not understanding consultant over the phone, especially cancer issues that caused panic,” “diagnosis is shocking and unexpected”
		Telephone triage provided reassurance: “Doctor over the phone was able to put my mind at rest"

## Discussion

Patients are generally satisfied with the telephone triage consultation implemented for the 2WW head and neck cancer referral process during the pandemic. A range of patient satisfaction aspects was rated highly, including being understood over the phone (Q1), duration of the consult (Q7), receiving adequate information (Q8) and feeling involved in their own care (Q9). Comparatively, these results are not significantly different from those who went for an initial face-to-face consultation.

In terms of accessibility to health services (Q3), a telephone consultation shines by increasing accessibility for those geographically isolated or housebound and provides convenience for those who have commitments at work or at home [19]. In our qualitative analysis, patients were understanding with regards to the need to socially distance and there were praises heaped for the increased convenience offered by the triage service. Yet, the quantitative mean score of 3.67 out of 5 was in contrast and significantly lower than the 4.29 for those who attended the face-to-face consultations. In our thematic analysis, one logical explanation could be due to perceived personal and organisational factors [20]. Individual patients’ perceptions of their healthcare needs may be different from that of healthcare professionals, and in this case, not being examined and a reduced interaction with the clinician over the phone may be interpreted as reduced access to their perception of 2WW suspected cancer services. Patients may also perceive additional organisational barriers using the triage pathway in terms of needing to arrange an additional appointment at a later time, hindering overall accessibility.

A significant finding of our study was that patients were satisfied with the relational aspects (Q4-6) of their care during the telephone consultation with the clinician, despite existing studies showing that the lack of visual cues and weaker rapport building, including social speech compared to their face to face counterparts, may have implications for the effective development of long-term relationships [19]. While credit must go directly to the specialists in our hospital, on a larger scale this could also reflect a positive trend in the overall NHS direction to continue investing in and developing projects in telemedicine while supporting clinicians to make the best use of these opportunities [21]. In fact, the positive patient perception of a telephone consultation (Q2) may have been built up by the overall increased use of telemedicine over the past decades, such as in general practice (GP). Even prior to the COVID-19 pandemic, 30% of all GP consultations are via telephone, video or online [22]. Since the pandemic, this has shifted to about 70%, and other surgical specialties, including orthopaedics [23] and maxillofacial surgery [24], have also begun utilising remote consultations. This study adds to the increasing evidence base that patients are satisfied with such a modality of clinical consult.

As for the effectiveness of the telephone triage (Q10), patients in our study perceived this to be between neutral and satisfied, which is significantly lower than the reported satisfaction for the patients in the F2F group (mean of 3.63 versus 4.75 out of 5). From our qualitative analysis, some of this dissatisfaction are intrinsic to the triage process, for example, patients expecting a physical examination or being told to return for a further appointment, while another source of dissatisfaction may be related to the outcome of the consult, a few patients felt that it was ineffective in addressing their needs. On the extreme end, one patient in our study reported having to seek a second opinion from a private specialist as she was anxious about the outcome of not being seen. A key theme in our analysis is that this consult also serves to provide reassurance for patients. Two-week wait appointments can be anxiety inducing, and one study found that 77.2% of patients were anxious to varying degrees about their referral to a tertiary head and neck cancer unit [25].

While it can be acknowledged that some patients are not necessarily reassured even in a face-to-face setting, nonverbal communication is essential alongside verbal messages to influence patients’ perception of a conversation and trust in the clinician [26]. Nonverbal messages are lacking in a telephone consultation, and effective communication regarding the topic of cancer can be hindered both ways: clinicians may not be able to express themselves as effectively, and some patients found their ability to communicate information hindered by a lack of visual/physical cues.

Other surgical specialties have reported much higher rates of patient satisfaction with the implementation of remote consultations during a similar time period, to the extent that 83% of patients attending a British general maxillofacial clinic [24] and 93% from a larger group of American orthopaedics clinic patients [27] would be willing to attend a similar remote consultation again. Our modest number of 55% may in part reflect the unique challenges of delivering a 2WW cancer triage service over the phone. First, 100% of our patients are new to the service, compared to 6.4% and 11.2% in the maxillofacial and orthopaedics clinic, respectively. Next, our patient group comprised suspected cancer patients, who are less likely to be present in either of the other two clinics. Interestingly, oral oncology patients made up a significant proportion of those who were unwilling to engage in a future phone consultation in the maxillofacial clinic study [24]. Yet, this may indicate that there are still ways we can continue to improve this service and learn from other specialties. For example, the use of video conferencing was successful in the orthopaedics study [27] and this could be explored in our setting.

The main limitation of this study is its design which compares patient satisfaction of a triage telephone consultation with a full initial face-to-face appointment. While this may not be a like for like comparison, the authors felt that an initial face-to-face appointment would provide the best yardstick for evaluating the new telephone triage consultation, to uncover themes behind the pros and cons of each modality, rather than evaluating the telephone triage consultation in isolation. With a 57.3% response rate, this study is also vulnerable to nonresponse bias, which could reduce the external validity of our custom patient satisfaction questionnaire. Finally, there is an element of recall bias, as participants were phoned four to six months after their initial consultation. In this context, some participants may have had further appointments with our service and may not accurately provide feedback on their initial experience.

Moving forwards, further studies can explore the effects of age and cancer diagnosis on patient satisfaction. Patient focus groups can also gather better qualitative data which may produce themes outwith the scope of a single broad open-ended question. Ultimately, on whether this telephone triage consultation should stay beyond the pandemic, patient satisfaction is only one component of this decision, and further studies including those exploring the efficacy of the risk calculator used, hospital resource allocation and clinician satisfaction with the process would also be instrumental in contributing to the overall decision.

## Conclusions

Overall, patient satisfaction for the initial telephone triage is positive, with satisfaction rates maintained for multiple aspects of care, and is comparable to that of a face-to-face consultation. When employed in a pandemic, this study supports the use of the telephone triage system from the patient’s perspective. However, some of its main advantages, for example, increased accessibility, are not as pronounced as expected, and there is a degree of perceived inefficacy of the telephone triage process particularly with the lack of physical examination. Some patients also felt that such a telephone consult increased their fear and anxiety over a possible cancer diagnosis. Beyond the pandemic, patient satisfaction response is mixed on whether the telephone triage system should stay for the foreseeable future.
